# Association Between MRI Findings of Facet Arthropathy and Synovitis With Health-Related Outcome and Pain Scores Following Therapeutic Lumbar Facet Injections

**DOI:** 10.1155/2024/7889539

**Published:** 2024-10-18

**Authors:** Michael S. Green, Michele Van Hal, Matthew Onimus, Christopher R. Hoffman, Dhruv K. C. Goyal, Valeria Potigailo, Khuram S. Kazmi

**Affiliations:** ^1^Department of Anesthesiology and Perioperative Medicine, Thomas Jefferson University, Sidney Kimmel Medical College, 111 S 11th Street, Philadelphia 19107, Pennsylvania, USA; ^2^Department of Anesthesiology, Drexel University College of Medicine, 2900 W Queen Ln, Philadelphia 19129, Pennsylvania, USA; ^3^Department of Anesthesiology, Rowan School of Osteopathic Medicine/Virtua Health System, 113 Laurel Rd, Stratford 08084, New Jersey, USA; ^4^Department of Radiology-Neuroradiology, University of Colorado School of Medicine, 13001 E 17th Pl, Aurora 80045, Colorado, USA; ^5^Department of Radiology, Cooper Medical School of Rowan University, 401 Broadway, Camden 08103, New Jersey, USA

## Abstract

Low back pain is a common complaint among adults. The facet joint is a major source of lumbar pain, and therapeutic facet injections have gained popularity as a minimally invasive treatment option. In addition, magnetic resonance imaging (MRI) utilization for diagnosing low back pain has increased significantly over the past few decades. Facet synovitis is an entity characterized by edema and inflammatory changes affecting the facet joints, adjacent bone marrow, and surrounding soft tissues. Although its underlying etiology remains poorly understood, recent reports suggest a high incidence in patients with arthropathy and arthritis. It is essential to explore potential correlations between specific MRI findings and outcomes after lumbar facet injections. This investigation is particularly relevant for facet synovitis, given its inflammatory nature and the common use of anti-inflammatory agents in facet injections. We investigated associations between MRI evidence of facet arthropathy and/or synovitis and the degree of improvement in health-related outcome and pain scores after therapeutic facet injections. The review was conducted on patients who received bilateral therapeutic facet injections, excluding those with prior lumbar spinal surgery or transitional segments. Facet arthropathy and synovitis were assessed on MRI by two neuroradiologists, and postprocedure outcomes such as pain and function were compared using univariate and multivariate analyses based on MRI findings. Our retrospective review indicates that patients receiving facet injections experience greater mean reduction in daily activity and workability burden scores from back pain when facet synovitis is a known portion of their pathology. The authors pose that further study could help identify patient populations that are the best candidates for therapeutic intervention. This may ultimately improve delivery of care, cost efficacy, and patient satisfaction.

## 1. Introduction

Low back pain is a common complaint among adults, with a majority of individuals experiencing these symptoms at some point in their lives. The facet joint is known to be a major source of lumbar pain, with prior studies showing a prevalence of approximately 27%–40% in the United States [1]. Low back pain attributed to the facet joints is known as “facet syndrome” and presents with some nonspecific symptoms including lower back pain, soreness, and stiffness. The condition typically worsens with extension of the spine or prolonged periods of sitting or standing and improves with movement throughout the day. As a minimally invasive treatment option, therapeutic facet injections have gained popularity in recent years. There has been tremendous growth in the utilization of this treatment option to address symptoms of lumbar pain over the past 2 decades; consequently, among Medicare beneficiaries, the annual growth rate from 2000 to 2011 was approximately 13.6% [2]. Over a similar time period, there has also been a sizeable increase in the utilization of magnetic resonance imaging (MRI) in the diagnosis of low back pain. From 1999 to 2008, the annual rate of growth of MRI as an imaging modality under these circumstances was 9% [3]. There are well-recognized imaging findings of facet osteoarthritis, including hypertrophic osteophytosis, facet joint effusion, subchondral signal changes, and synovial cyst formation.

More recently, however, an entity known as “facet synovitis” has been recognized. To our knowledge, this term was first coined in the published literature in 2008 by Czervionke and Fenton; however, the condition is now much more recognized due to the routine use of fat-saturated T2 (short tau inversion recovery [STIR]) sequences [4]. Facet synovitis refers to edema and inflammatory changes involving the facet joints, their adjacent bone marrow, and the surrounding soft tissues. The underlying etiology and pathophysiology behind this condition remain poorly understood. There is a recent report which describes a high incidence of facet synovitis in patients with enthesitis-related arthropathy, a subtype of juvenile rheumatoid arthritis, suggesting an underlying inflammatory component [5]. However, there remains little, if any, pathologic correlation between the two conditions.

Given the widespread use of both lumbar MRI and facet injections in the management of low back pain, as well as the well-recognized MRI findings of facet arthritic disease and synovitis, it would be helpful to determine if there was any correlation between certain MRI findings and outcomes following lumbar facet injections. This is particularly relevant for facet synovitis given its inflammatory nature and the standard use of anti-inflammatory agents contained in facet injections.

The purpose of this study was to determine if there is any association between MRI evidence of facet arthropathy and/or synovitis and the degree of improvement in health-related outcome and pain scores after therapeutic facet injections in the lumbar spine.

## 2. Materials and Methods

Upon obtaining approval from the Drexel University Institutional Review Board (IRB), a retrospective review was conducted on patients who received therapeutic facet injections from L4–L5 level at a single, teaching hospital between March 2013 and May 2015. Patients who received a lumbar spine MRI performed at our institution were included in the final cohort. Anyone who had a history of prior lumbar spinal surgery with spinal hardware was excluded from the analysis since (1) postsurgical soft tissue and bony signal changes can persist for months or years after surgery, thereby potentially obscuring or mimicking facet disease, and (2) spinal hardware may result in imaging artifacts in the tissues that typically need to be assessed for facet disease.

**Figure 1:**
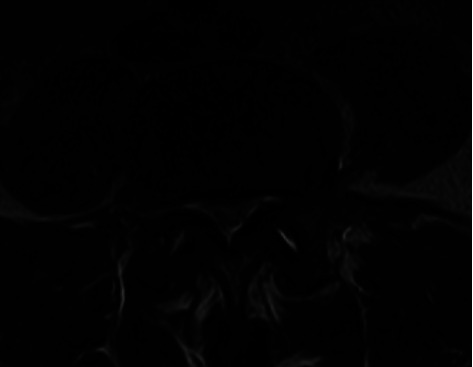
Axial T2-weighted image at the L4–L5 level demonstrates enlarged facet joints bilaterally with marginal osteophytes and joint effusions.

**Figure 2:**
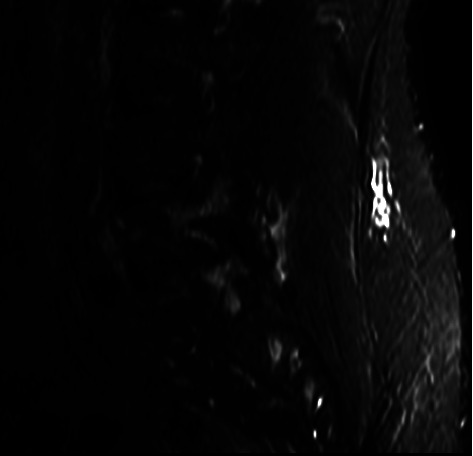
Sagittal short tau inversion recovery (STIR) sequence demonstrates increased signal within the bone marrow and soft tissues surrounding the L5–S1 facet joint.

Each MRI examination was reviewed by two fellowship-trained neuroradiologists, who did not perform any of the injections, to detect arthritic changes, including hypertrophy secondary to marginal osteophyte formation with or without subchondral signal changes, synovial cyst formation, or facet joint effusion (Figure 1). Each imaging study was also screened for the presence or absence of facet synovitis, defined as the presence of edema within the soft tissues or bone marrow surrounding the facet joints (Figure 2). Both facet arthropathy and facet synovitis were documented for each individual.

Each neuroradiologist was blinded to the procedural results at the time they reviewed the MRI examinations. Consequently, if there were any discrepancies in MRI interpretations, the two neuroradiologists reviewed the scans a second time together in order to reach a consensus regarding the presence or absence of arthritic changes or synovitis. If a consensus could not be reached, those patients were excluded from the final sample. Lastly, patients who had transitional segments where L4–L5 could not be definitely identified were also excluded.

Each facet injection was performed by a single operator, a fellowship-trained interventional pain management specialist in practice since 2005. The operator was not always aware of the MRI findings prior to each injection. All of the procedures were performed bilaterally at L4–L5, under fluoroscopic guidance in order to target the proper location. Localization was based on the anatomic position of the needle on the fluoroscopic image and a “give in” feeling that was reported. The pain management specialist did not inject intra-articular contrast injection, as a matter of routine practice. Injections were performed utilizing a mixture of local anesthetic (1.75 mL of 0.5% lidocaine) and corticosteroid (10 mg of triamcinolone) for a total of 2 mL of solution. Sedation was not used for any of the injections performed.

Procedural records and office notes were then reviewed by a fourth physician, neither the pain management physician performing the procedures nor the neuroradiologists reviewing the MRI studies, for various demographics, including age, sex, and number of lumbar injections performed. In addition, pre- and postprocedure health-related outcome scores—including daily activity level, sleep quality, and workability—and average pain scores were documented for each individual and based on a 0–10 scale of increasing severity. Preprocedure outcome and pain score had been recorded by the patient on the day of the procedure, while postprocedure outcome and pain scores were recorded on the final follow-up visit, typically 6–8 weeks, or longer, after the procedure. Almost all, if not all, facet injections were preceded (4–8 weeks prior) by lumbosacral transforaminal injections in order to exclude a radicular component of the pain.

Upon completion of data collection, patients were split into groups based on the presence of MRI findings of facet arthropathy or not, and the presence of facet synovitis or not. Preprocedure, postprocedure, and delta (post minus pre) daily activity, sleep quality, workability, and pain scores were compared between groups using univariate analysis—independent-samples (Student's) *t*-test or Mann–Whitney *U* test. Multiple linear regression was performed to determine whether the presence of facet arthropathy or synovitis on MRI imaging predicted postprocedure outcome scores, controlling for a variety of independent variables including age, sex, number of levels injected, and the presence of either facet arthropathy or synovitis on MRI. Subsequently, patients in facet arthropathy and synovitis groups were further subdivided into a subanalysis to determine whether unilateral or bilateral MRI characteristics influenced postprocedure outcomes, using univariate—specifically Kruskal–Wallis H test with Dunn multiple pairwise comparison post hoc analysis—and multivariate analyses. All statistical analyses were performed with the Statistical Package for the Social Sciences (SPSS) Version 25 (IBM Corporation, Armonk, New York).

## 3. Results

One hundred and fifty-six patients who had facet injections at L4–L5 were initially screened. Ninety-one patients without any of the exclusion criteria discussed above were included in the final analysis. The mean age of the cohort was 58 (standard deviation [SD] = 12.0) years and there were 33 (36.3%) males and 58 (63.7%) females included in the study. A total of 49 (53.8%) patients were found to have radiographic MRI findings of facet arthropathy, with 13 (14.3%) and 36 (39.5%) of these individuals demonstrating unilateral and bilateral characteristics, respectively. A total of 16 (17.5%) patients were found to have MRI characteristics of facet synovitis, with 9 (9.9%) and 7 (7.6%) individuals exhibiting unilateral and bilateral findings, respectively. All patients who demonstrated evidence of facet synovitis also demonstrated characteristic imaging findings of facet arthropathy. As such, 33 (36.3%) patients were found to have facet arthropathy alone on MRI. Most individuals received only one (38 patients, 41.8%) or two (29 patients, 31.9%) facet injections for symptomatic relief. Demographics and mean preprocedure, postprocedure, and delta outcome and pain scores among the cohort can be found in Table 1.

In the primary analysis, there were no significant differences between facet arthropathy and facet synovitis groups based on univariate analysis. On multivariate analysis, having facet synovitis (either unilateral or bilateral) characteristics on MRI significantly predicted lower average burden scores from low back pain for postprocedure daily activity (*β*-coefficient = −1.929 [−3.845, −0.012]; *p*=0.049) and workability (*β*-coefficient = −2.901 [−5.254, −0.549]; *p*=0.016), but not sleep quality or pain scores (Table 2).

In the subanalysis, bilateral synovitis characteristics were associated with a significantly greater mean reduction in daily activity burden (−5.00 [SD = 1.79]) than those with unilateral findings (−1.67 [SD = 3.28]) or no evidence (−1.66 [SD = 3.07]) of facet synovitis. Multivariate analysis demonstrated that presence of bilateral facet synovitis predicted lower average postprocedure daily activity (*β*-coefficient = −3.971 [−6.712, −1.230]; *p*=0.005) and workability (*β*-coefficient = −4.479 [−8.092, −0.867]; *p*=0.016) burden scores from back pain and pain (*β*-coefficient = −2.018 [−3.892, −0.144]; *p*=0.035) scores than individuals with no evidence of facet synovitis on MRI (Table 3).

## 4. Discussion

The term “facet syndrome” first appeared in the published literature in the early 1970s and the potential utilization of facet joint injections as a therapeutic modality was described not long after that in the early 1980s [6, 7]. There are data in the literature for the use of these procedures for both the diagnosis and therapy of the lumbar facet joint in cases of low back pain. The evidence for “diagnostic facet joint techniques is categorized as Level I or Level II-1” [1]. That same article describes the evidence for therapeutic “lumbar intraarticular injections as Level III (limited) with a recommendation of 2C/very weak or recommendation not to be provided” [1]. The MRI findings of lumbar facet disease are also well recognized and have been extensively described [8, 9].

Given the relatively common utilization of both MRI and lumbar facet injections in the diagnosis and management of facet joint–mediated lower back pain, it would be beneficial to identify which imaging characteristics could predict improvement in health-related outcome and pain scores in patients who receive therapeutic lumbar facet injections for symptomatic resolution; therefore, the purpose of this study was to further characterize this association to improve outcomes in patients with facet syndrome.

Cross-sectional imaging has been studied extensively with variable results. In general, however, there are more studies that have failed to show a correlation between facet imaging findings and response to injections, than studies that have demonstrated a positive correlation [10–13]. In particular, a study by Stojanovic et al. attempted to look at the potential predictive value of MRI in these situations [14]. While the authors noticed an association between MRI findings and the response to lumbar facet injections, the findings were weak and not significant enough to elicit widespread practice changes. Other studies, including a 2006 study by Gorbach et al. and a 2017 study by Hofmann et al. failed to show any correlation between MRI features and pain outcome measures [15, 16]. Most of the studies have looked at anatomic changes of the facet joints, which can be problematic in assessing the association between imaging characteristics and pain response following therapeutic facet injections. First, the anatomic changes of facet arthropathy are often chronic and may not account for the patient's current symptoms. Also, multiple studies have shown, at best, moderate interobserver and intraobserver reliability for computed tomography (CT) and MRI in rating facet joint degeneration in the lumbar spine [17, 18]. Our study attempted to minimize these concerns by using a consensus of two experienced MRI readers. Furthermore, Lee et al. were able to compare CT and MRI with histologic examination of the facet joints and found that the imaging significantly underestimated the degree of facet degeneration seen at histology, which could also explain why anatomic findings alone are inadequate [19].

To our knowledge, this is the first study that has attempted to elucidate whether a correlation between facet synovitis and response to facet interventions exists. Facet synovitis has become more widely recognized in the past decade with the now routine use of fat-saturated T2-weighted imaging of the lumbar spine [4, 20]. Facet synovitis is thought to represent active inflammation and has been shown to correlate with a patient's site of pain [4]. Therefore, it would stand to reason that patients with synovitis may have a better response to combined anesthetic and anti-inflammatory injections than patients with purely anatomic facet arthropathy features. The results of our study demonstrated that the presence of facet synovitis predicted a greater reduction in daily activity (*β*-coefficient = −1.929 [−3.845, −0.012]; *p*=0.049) and workability (*β*-coefficient = −2.901 [−5.254, −0.549]; *p*=0.016) burden scores from low back pain on multivariate analysis. When considering the results of our subanalysis, having bilateral facet synovitis, specifically, predicted a greater reduction in daily activity (*β*-coefficient = −3.971 [−6.712, −1.230]; *p*=0.005) and work ability (*β*-coefficient = −4.479 [−8.092, −0.867]; *p*=0.016) burden and pain scores (*β*-coefficient = −2.018 [−3.892, −0.144]; *p*=0.035) on postprocedure measurements compared to having purely facet arthropathy. This may be in part due to the fact that every patient received bilateral facet injections, and so patients with bilateral facet synovitis may have gained the most benefit in our particular cohort of patients.

Other studies in the published literature have demonstrated similar findings to the ones in our study. Koh et al. attempted to utilize nuclear medicine imaging to predict short-term outcomes after medial branch block [21]. They prospectively analyzed 33 patients with suspected facet disease and imaged each patient with bone scintigraphy with single photon emission CT (SPECT). Utilizing the visual analog score, the authors found that patients who had positive SPECT scans demonstrated a significantly better response to ultrasound-guided medial branch block than SPECT-negative patients. They noted a high sensitivity of 96% at 2 weeks and 100% at 4 weeks. Specificity, however, was much lower at 50% and 45% at 2 and 4 weeks, respectively. In addition, the authors found no significant difference between the SPECT-positive and SPECT-negative groups in terms of a change in their Oswestry disability index scores. Overall, they concluded that SPECT was useful in diagnosing facet disease and determining the exact location for a medial branch block to be attempted. An earlier study by Dolan et al. also showed a correlation between uptake on SPECT and short-term outcomes following lumbar facet injections [22]. Each of these studies evaluated uptake on bone scintigraphy, which is thought to represent a more active process [21, 22]. This is similar to the theory of facet synovitis on MRI [4]. There are significant benefits to utilizing MRI over bone scintigraphy as a screening tool to assess for active inflammation of the facet joints, including widespread availability, patient comfort, and no ionizing radiation. In fact, it is likely that MRI has already been performed in many patients who are being evaluated for facet injection, as was the case in our cohort.

There are several limitations of our study, the most important one being a small sample size of patients included in the cohort. Since there were only 91 subjects, 16 with evidence of facet synovitis and 7 with bilateral characteristics, our results should be taken with caution as a larger sample size would have increased the strength of our findings. Future prospective investigations with larger numbers, including both patients who received unilateral and bilateral injections, would be useful to further delineate the influence of imaging characteristics as predictors of improved outcomes following therapeutic lumbar facet injections. In addition, given that this was a retrospective review of existing data, there are some variables which were not strictly controlled for, such as the exact time in which postprocedure pain scores were obtained or whether the pain management physician had knowledge of the MRI findings prior to intervention. Another potential limitation is that contrast was not injected to confirm that an intra-articular location was achieved. This was our pain management specialist's routine practice and could not be altered retrospectively; therefore, we have considered these to be facet injections, rather than joint injections. While this may limit our study's assessment of joint pain and the response to joint injections, it may help explain the relatively strong response in the few patients with bilateral facet synovitis. The imaging findings in facet synovitis are thought to represent inflammation and are often prominent in the surrounding soft tissues. It might stand to reason that a therapeutic injection in the region of the facet joint may provide relief, regardless of whether or not it was definitively intra-articular. Also, since our data were obtained from existing procedural records, we did not have any control subjects as comparisons; as such, all patients received therapeutic injections and none of them received placebo or conservative therapy. Lastly, a limitation of the study is that it only examines the presence or absence of facet joint arthritis without assessing the degree of its severity and how that might impact the outcome of the injection.

## 5. Conclusions

The results of our study demonstrate that patients with imaging characteristics of facet synovitis on MRI demonstrated greater improvement in health-related outcome and pain scores following lumbar facet injections on univariate and multivariate analyses. Furthermore, patients with bilateral synovitis appeared to have gained the most benefit from this therapeutic modality. Our study is the first to test the association between imaging characteristics of facet synovitis and response to therapeutic facet injections in patients with lumbar facet syndrome. As such, this should serve as an initial, exploratory study. Future prospective studies containing larger sample sizes are warranted in order to further delineate the strength of imaging characteristics as predictors of health-related outcome improvement and pain reduction in patients who receive therapeutic lumbar facet injections.

## Figures and Tables

**Table 1:** Demographics of cohort.

Overall (*n* = 91)	
Age	58.0 (12.0)

*Sex*	
M	33 (36.3%)
F	58 (63.7%)

*Number of lumbar injections*	
1	38 (41.8%)
2	29 (31.9%)
3	9 (9.9%)
4	15 (16.5%)

*Daily activity*	
Pre	8.44 (2.17)
Post	6.60 (3.30)
Delta	−1.89 (3.11)

*Sleep quality*	
Pre	8.22 (2.58)
Post	6.63 (3.54)
Delta	−1.52 (3.38)

*Workability*	
Pre	7.95 (3.06)
Post	5.76 (3.98)
Delta	−2.12 (3.88)

*Pain score*	
Pre	8.18 (1.75)
Post	6.46 (2.67)
Delta	−1.71 (2.18)

*Facet arthropathy?*	
No	42 (46.2%)
Yes	49 (53.8%)
Unilateral	13 (14.3%)
Bilateral	36 (39.5%)

*Facet synovitis?*	
No	75 (82.5%)
Yes	16 (17.5%)
Unilateral	9 (9.9%)
Bilateral	7 (7.6%)

*Note:* Categorical and continuous values are represented as *n* (%) and mean (standard deviation), respectively. Delta = postoperative outcome minus preoperative outcome score.

**Table 2:** Outcome comparisons between facet arthropathy/synovitis groups.

		Facet arthropathy			Facet synovitis			Multivariate analysis: *β*-coefficient (95% CI), *p*^2^
		No arthropathy (*n* = 42)	Arthropathy (*n* = 49)	*p* ^1^	No synovitis (*n* = 75)	Synovitis (*n* = 16)	*p* ^1^	
Daily activity	Pre	8.45 (2.09)	8.44 (2.26)	0.974	8.41 (2.26)	8.60 (1.68)	0.972	FA: 1.285 (−0.372, 2.942), 0.127
	Post	6.43 (3.27)	6.75 (3.36)	0.648	6.76 (3.19)	5.88 (3.81)	0.505	
	Delta	−2.02 (3.54)	−1.77 (2.71)	0.699	−1.66 (3.07)	−3.00 (3.18)	0.148	FS: −1.929 (−3.845, −0.012), 0.049∗

Sleep quality	Pre	8.05 (2.68)	8.37 (2.51)	0.563	8.12 (2.73)	8.69 (1.74)	0.632	FA: 1.567 (−0.213, 3.347), 0.084
	Post	6.33 (3.50)	6.90 (3.59)	0.455	6.69 (3.50)	6.38 (3.86)	0.843	
	Delta	−1.56 (3.51)	−1.48 (3.31)	0.910	−1.34 (3.25)	−2.31 (3.94)	0.641	FS: −1.470 (−3.432, 0.492), 0.140

Workability	Pre	8.00 (2.92)	7.91 (3.22)	0.896	7.85 (3.22)	8.50 (2.03)	0.834	FA: 1.889 (−0.124, 3.903), 0.066
	Post	5.64 (3.93)	5.87 (4.07)	0.788	6.03 (3.98)	4.47 (3.89)	0.117	
	Delta	−2.22 (4.22)	−2.02 (3.60)	0.816	−1.74 (3.72)	−4.07 (4.27)	0.077	FS: −2.901 (−5.254, −0.549), 0.016∗

Pain score	Pre	8.07 (1.79)	8.27 (1.74)	0.602	8.24 (1.75)	7.88 (1.78)	0.382	FA: 0.829 (−0.373, 2.031), 0.174
	Post	6.17 (2.95)	6.71 (2.41)	0.332	6.64 (2.61)	5.62 (2.87)	0.165	
	Delta	−1.90 (2.36)	−1.55 (2.03)	0.444	−1.60 (2.07)	−2.25 (2.65)	0.398	FS: −1.142 (−2.510, 0.226), 0.101

*Note:* Outcomes are reported as mean (standard deviation). Delta = postoperative outcome minus preoperative outcome score. *p*^1^ = independent-samples (Student's) *t*-test or Mann–Whitney *U* test. *p*^2^ = multiple linear regression (dependent variable postoperative outcome).

Abbreviations: FA = facet arthropathy, FS = facet synovitis.

^∗^Statistical significance (*p* < 0.05).

**Table 3:** Outcome comparisons between facet arthropathy/synovitis groups (subgroup analysis).

		Facet arthropathy				Facet synovitis				Multivariate analysis: *β*-coefficient (95% CI), *p*^2^
		No arthropathy (*n* = 42)	Unilateral arthropathy (*n* = 13)	Bilateral arthropathy (*n* = 36)	*p*	No synovitis (*n* = 75)	Unilateral synovitis (*n* = 9)	Bilateral synovitis (*n* = 7)	*p*	
Daily activity	Pre	8.45 (2.09)	8.31 (2.50)	8.49 (2.20)	0.982	8.41 (2.26)	8.44 (2.01)	8.83 (1.17)	0.995	FA unilateral: 0.755 (−1.452, 2.961), 0.498 FA bilateral: 1.308 (−0.458, 3.074), 0.144 FS unilateral: −0.459 (−2.839, 1.920), 0.702 FS bilateral: −3.971 (-6.712, −1.230), 0.005∗
	Post	6.43 (3.27)	6.62 (3.45)	6.80 (3.38)	0.819	6.76 (3.19)	6.78 (4.09)	4.71 (3.35)	0.277	
	Delta	−2.02 (3.54)	−1.69 (3.57)	−1.79 (2.37)	0.657	−1.66 (3.07)	−1.67 (3.28)	−5.00 (1.79)	0.025∗	

Sleep quality	Pre	8.05 (2.68)	8.31 (3.01)	8.39 (2.36)	0.706	8.12 (2.73)	8.44 (2.13)	9.00 (1.15)	0.891	FA unilateral: 1.000 (−1.379, 3.379), 0.405 FA bilateral: 1.718 (−0.215, 3.652), 0.081 FS unilateral: −0.687 (−3.223, 1.848), 0.591 FS bilateral: −2.354 (−5.088, 0.380), 0.091
	Post	6.33 (3.50)	6.62 (3.66)	7.00 (3.61)	0.496	6.69 (3.50)	6.89 (4.11)	5.71 (3.73)	0.632	
	Delta	−1.56 (3.51)	−1.69 (3.90)	−1.40 (3.12)	0.725	−1.34 (3.25)	−1.56 (4.16)	−3.29 (3.73)	0.441	

Workability	Pre	8.00 (2.92)	7.50 (3.68)	8.06 (3.08)	0.877	7.85 (3.22)	9.11 (1.36)	7.40 (2.70)	0.349	FA unilateral: 1.145 (−1.622, 3.912), 0.413 FA bilateral: 2.092 (−0.088, 4.272), 0.060 FS unilateral: −1.817 (−4.742, 1.108), 0.220 FS bilateral: −4.479 (−8.092, −0.867), 0.016∗
	Post	5.64 (3.93)	5.46 (3.97)	6.03 (4.15)	0.734	6.03 (3.98)	5.67 (4.39)	2.67 (2.25)	0.123	
	Delta	−2.22 (4.22)	−2.08 (4.48)	−2.00 (3.31)	0.779	−1.74 (3.72)	−3.44 (4.59)	−5.20 (3.83)	0.137	

Pain score	Pre	8.07 (1.79)	7.77 (2.20)	8.44 (1.54)	0.594	8.24 (1.75)	7.67 (2.12)	8.14 (1.35)	0.672	FA unilateral: 1.040 (−0.569, 2.649), 0.202 FA bilateral: 0.614 (−0.680, 1.908), 0.348 FS unilateral: −0.469 (−2.215, 1.278), 0.595 FS bilateral: −2.018 (−3.892, −0.144), 0.035∗
	Post	6.17 (2.95)	6.69 (2.69)	6.72 (2.34)	0.757	6.64 (2.61)	6.11 (3.30)	5.00 (2.31)	0.191	
	Delta	−1.90 (2.36)	−1.08 (2.90)	−1.72 (1.63)	0.276	−1.60 (2.07)	−1.56 (2.96)	−3.14 (2.04)	0.161	

*Note:* Outcomes reported as mean (standard deviation). Delta = postoperative outcome minus preoperative outcome score. *p*^1^ = Kruskal–Wallis *H* test with Dunn multiple pairwise comparison post hoc analysis. *p*^2^ = multiple linear regression (dependent variable postoperative outcome).

^∗^Statistical significance (*p* < 0.05).

## Data Availability

The data used to support the findings of this study are available from the corresponding author upon reasonable request.
